# Current status and outcomes of patients developing PSA recurrence after prostatectomy who were treated with salvage radiotherapy: a JROSG surveillance study

**DOI:** 10.1093/jrr/rrv027

**Published:** 2015-04-24

**Authors:** Takashi Mizowaki, Manabu Aoki, Katsumasa Nakamura, Atsunori Yorozu, Masaki Kokubo, Katsuyuki Karasawa, Takuyo Kozuka, Nobuaki Nakajima, Keisuke Sasai, Tetsuo Akimoto

**Affiliations:** 1 Department of Radiation Oncology and Image-applied Therapy, Graduate School of Medicine, Kyoto University. 54 Shogoin Kawahara-cho, Sakyo-ku, Kyoto, 606-8507, Japan; 2 Department of Radiology, The Jikei University School of Medicine, 3-25-8 Nishishinbashi, Minato-ku, Tokyo, 105-8461, Japan; 3 Department of Clinical Radiology, Graduate School of Medical Sciences, Kyushu University, 3-1-1 Umade, Higashi-ku, Fukuoka, 812-8582, Japan; 4 Department of Radiology, National Tokyo Medical Center, 2-5-1 Higashigaoka, Meguro-ku, Tokyo, 152-8902, Japan; 5 Department of Radiation Oncology, Kobe City Medical Center General Hospital, 2-1-1 Minatojimaminamimachi, Chuo-ku, Kobe, 650-0047, Japan; 6 Department of Radiology, Tokyo Metropolitan Cancer and Infectious diseases Center, Komagome Hospital, 3-18-22 Honkomagome, Bunkyo-ku, Tokyo, 113-8677, Japan; 7 Department of Radiation Oncology, Cancer Institute Hospital, 3-8-31 Ariake, Kouto-ku, Tokyo, 135-8550, Japan; 8 Department of Radiology, Shizuoka General Hospital, 4-27-1 Kitaando, Aoi-ku, Shizuoka, 420-8527, Japan; 9 Department of Radiology, Juntendo University School of Medicine, 3-1-3 Hongo, Bunkyo-ku, Tokyo, 113-8431, Japan; 10 Department of Radiation Oncology and Particle Therapy, National Cancer Center Hospital East, 6-5-1 Kashiwanoha, Kashiwa City, Chiba, 277-8577, Japan

**Keywords:** salvage radiotherapy, prostate cancer, PSA recurrence, radical prostatectomy

## Abstract

The conditions and outcomes of Japanese patients with prostate cancer who developed PSA failure after radical prostatectomy (RP), and who were treated via salvage radiotherapy (S-RT), were surveyed. Clinical data on S-RT were gathered in questionnaires completed by facilities participating in the Japanese Radiation Oncology Study Group. S-RT was defined as external-beam radiotherapy delivered to the prostate beds of patients with prostate cancer who had eventually developed PSA failure, although their PSA values had at one stage attained levels <0.2 ng/ml following RP. Hormonal therapy was combined with S-RT in ∼40% of cases. Outcomes were evaluated in 186 cases treated via S-RT alone. The nadir PSA level after RP, and the level upon initiation of S-RT, were 0.0135 ng/ml and 0.292 ng/ml, respectively. The median period between RP and S-RT was 18.6 months. The median follow-up period was 58 months. The 5-year PSA recurrence–free survival (PRFS) and clinical failure–free survival (CFFS) rates were 50.1% (95% CI: 42.8–57.9%) and 90.1% (95% CI: 86.4–95.7%), respectively. PRFS was significantly superior in patients with PSA values ≤0.3 ng/ml upon initiation of S-RT than in those with PSA values >0.3 ng/ml (57.5% vs 40.5%, *P* = 0.027). In Japan, hormonal therapy is combined with S-RT in ∼40% of cases. The 5-year PRFS and CFFS rates of cases treated via S-RT alone were 50.1% and 90.1%, respectively. A PSA value of 0.3 ng/ml served as a significant cut-off for prediction of PRFS.

## INTRODUCTION

Radical prostatectomy (RP) is one of the principal treatment modalities for localized prostate cancer. However, about 20–30% of patients with localized disease treated via RP eventually experience recurrences [1]. Of such cases, some patients exhibit clinical recurrence, including lymph node and bone metastases. However, most recurrences are prostate-specific antigen (PSA) recurrences, in which continuous rises in PSA levels are observed, without any evidence of clinical failure, after a very low PSA nadir value has been attained after RP. It has been reported that 34% of PSA-recurrent cases develop metastatic disease at a median time of 8 years after such PSA elevations, and die at a median time of 5 years from development of metastatic disease [1].

In patients exhibiting PSA recurrence, salvage radiotherapy (S-RT) is considered to be the only curative treatment. Therefore, S-RT has been widely used to treat those who have developed PSA recurrence after RP, although the impact thereof on survival remains under investigation by the Japan Clinical Oncology Group (JCOG) (the JCOG 0401 study [2]). The American Society for Radiation Oncology (ASTRO)/American Urological Association (AUA) guidelines strongly recommend that S-RT should be offered to patients exhibiting PSA or local recurrence after RP, if there is no evidence of distant metastatic disease [3]. However, no nationwide data on the outcomes of S-RT in Japan are available. Therefore, we conducted the present study to determine the actual conditions and outcomes of patients treated with S-RT upon the development of PSA recurrence after RP in Japan.

## METHODS

A registry was established by facilities participating in the Japanese Radiation Oncology Study Group (JROSG). Eligible cases were patients with localized prostate cancer who were treated via RP prior to 2005 and who received S-RT between January 2005 and December 2007 because of PSA failure. The cut-off PSA value for PSA failure was defined as 0.2 ng/ml based on the Guidelines for Clinical Practices of Prostate Cancer edited by the Japanese Urorogical Association [4], and the ASTRO/AUA guidelines [3]. S-RT featured external-beam radiotherapy delivered to the prostate beds of patients with prostate cancer who had eventually developed PSA failure after RP, although their PSA levels had once dropped below 0.2 ng/ml at some point after RP. Therefore, patients with minimum PSA values after RP (the PSA nadirs) of 0.2 ng/ml or higher were excluded from the present study. In addition, the interval between RP and S-RT was essentially required to be 6 months or longer. Patients in whom EBRT was delivered to the prostate bed earlier than 3 months after RP were considered to have received adjuvant radiotherapy, and their data were reported separately [5]. Because pre-operative and operative factors have been well studied [6, 7], this survey focused on the post-operative factors relating to S-RT in order to maximize the reliability of data from busy JROSG facilities.

PSA recurrence–free survival (PRFS) and clinical failure–free survival (CFFS) rates were calculated via the Kaplan–Meier method, commencing on the dates of initiation of S-RT. The statistical significances of observed differences in survival curves were estimated using the log-rank test. PSA recurrence developing after S-RT was defined as follows: The PSA level became re-elevated to 0.2 ng/ml or higher in patients in whom the PSA level had once dropped below 0.2 ng/ml after S-RT, or was 0.2 ng/ml or higher on the date of the first measurement of PSA level after S-RT if the PSA level had never fallen below 0.2 ng/ml. Cox's proportional hazard modeling was used to explore the predictive significance of factors associated with PRFS. The grading of each adverse event was based on the Common Terminology Criteria for Adverse Events (CTCAE) version 4.0; JCOG [8]. The Mann–Whitney U-test was used to compare the incidences of adverse events among patients treated with different radiation techniques or radiation doses. Statistical analyses were performed with the aid of GraphPad Prism 5.04 (GraphPad Software Inc., La Jolla, CA, USA) and StatView (ver. 5.0; SAS Institute Inc., Cary, NC, USA) software packages.

The present study was designed and conducted by the Urologic Oncology Subgroup of the JROSG. The study was also approved by the Institutional Review Boards of Kyoto University (Approval No.: E-1007) and Jikei University, and conducted in accordance with the dictates of the Helsinki Declaration.

## RESULTS

### Overview of the cases

Data on 371 cases treated in 38 facilities were sent to the JROSG registry between October 2011 and January 2012. Hormonal therapy was combined with S-RT in 151 patients (40.7% of all cases). In three cases, chemotherapy was added before or after S-RT. The rest of the cases were treated via S-RT alone. However, prognostic information was insufficient in 28 cases, and the PSA nadir value was higher than 0.2 ng/ml in a further 3 cases. Therefore, PRFS and CFFS were evaluated for the remaining 186 cases who met the criteria for S-RT and who were treated via S-RT alone. PSA doubling time was not included in the current analyses, because it was not reported in many cases.

### Characteristics of cases treated via S-RT alone

The characteristics of the 186 cases treated via S-RT alone are summarized in Table 1. The median patient age was 67 years. The nadir PSA level after RP and the PSA level at initiation of S-RT were 0.0135 ng/ml and 0.292 ng/ml, respectively. The median period between RP and S-RT was 18.6 (range: 3.8–80.5) months. In seven cases, S-RT was commenced between 3 and 6 months after RP (3.8–5.2 months).

**Table 1.** Characteristics of the S-RT–alone cases

Age (years)	Range: 49–82
	Median: 67
	Mean: 66
PSA nadir after RP (ng/ml)	Range: <0.0–0.191
	Median: 0.0135
	Mean: 0.032
PSA at initiation of S-RT (ng/ml)	Range: 0.02–3.63
	Median: 0.292
	Mean: 0.402
Period between RP and initiation of S-RT (months)	Range: 3.8–80.5
	Median: 18.6
	Mean: 24

PSA = prostate-specific antigen, RP = radical prostatectomy, S-RT = salvage radiotherapy.

### Details of S-RT

Of the 186 cases, computed tomography-based simulations were performed on 155, whereas X-ray simulations were performed on 31. The prostate bed, small pelvis and entire pelvis were irradiated in 176, 8 and 2 cases, respectively. Three-dimensional conformal radiation therapy (3D-CRT) with five portals or more, the four-field box technique, and an anterior–posterior opposing field, were used in 88, 96 and 2 cases, respectively. The X-ray energies were as follows: 6 MV in 6, 10 MV in 144, and >10 MV in 34 cases, respectively. The prescribed doses were >70 Gy, >65 Gy to ≤70 Gy, >60 Gy to ≤65 Gy, and ≤60 Gy in 6, 70, 105 and 5 patients, respectively. The cone-down technique was used in 71 cases, with boost plans applied after a median dose of 45.8 Gy (range: 30–60 Gy) had been delivered. A summary of the S-RTs applied is shown in Table 2.

**Table 2.** Summary of salvage radiotherapies

Treatment planning method		Number of cases
	CT-based plan	155
	X-ray simulation	31
Irradiated area		
	Prostate bed	176
	Small pelvis	8
	Whole pelvis	2
Radiation technique		
	Five or more fields	88
	Four fields	96
	Other	2
Total dose		
	>70 Gy	6
	>65 Gy to ≤70 Gy	70
	>60 Gy to ≤65 Gy	105
	≤ 60 Gy	5

CT = computed tomography.

### Oncological outcomes of S-RT

**Fig 1.**
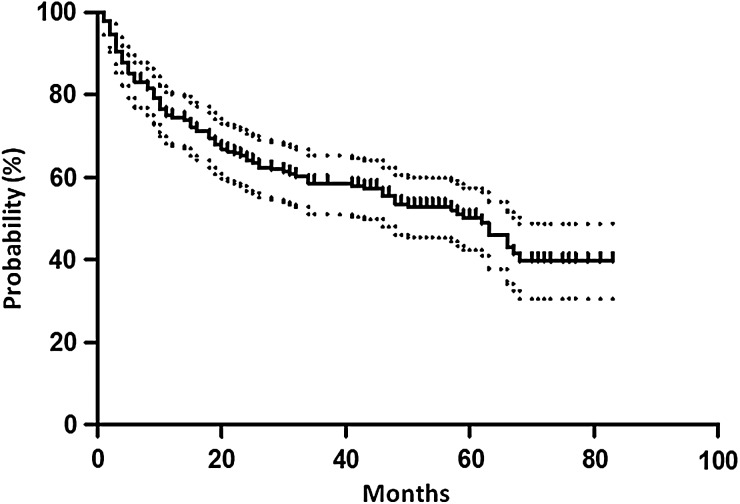
Kaplan–Meier curve with 95% confidence interval of PSA recurrence–free survival.

**Fig 2.**
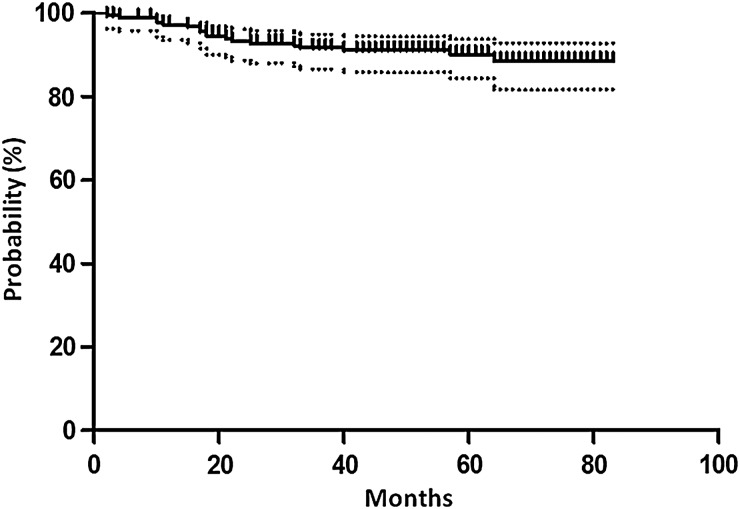
Kaplan–Meier curve with 95% confidence interval for clinical failure–free survival.

**Fig 3.**
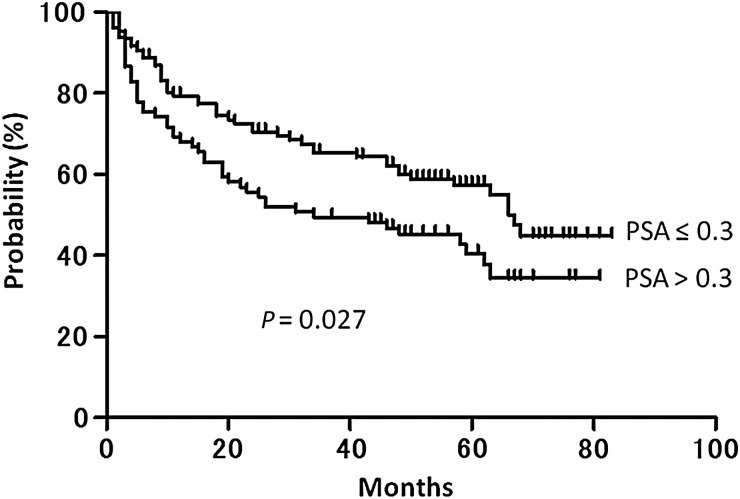
Kaplan–Meier curves for PSA recurrence–free survival, according to PSA level at initiation of salvage radiotherapy (PSA ≤ 0.3 ng/ml vs PSA > 0.3 ng/ml).

The median follow-up period was 58 months (range: 3–83 months). The 5-year PRFS and CFFS were 50.1% (95% CI: 42.8–57.9%) and 90.1% (95% CI: 86.4–95.7%), respectively (Figs 1 and 2). Of the PSA level at initiation of S-RT (the pre-S-RT PSA), the PSA nadir attained after RP, the period between RP and S-RT, radiation dose, and age, only the pre-S-RT PSA level significantly predicted PRFS upon both univariate and multivariate analyses (Table 3). The PRFS was significantly longer in patients with PSA values ≤0.3 ng/ml at initiation of S-RT than in those with PSA levels >0.3 ng/ml (57.5% vs 40.5%, *P* = 0.027) (Fig. 3). With other PSA cut-off values at S-RT, the differences were not statistically significant (*P* = 0.44, 0.051 and 0.21 for 0.2, 0.4 and 0.5 ng/ml, respectively).

**Table 3.** Univariate and multivariate analyses of factors predicting PSA failure–free survival upon Cox's proportional hazard modeling

	Univariate			Multivariate		
Factor	HR	95% CI	*P*	HR	95% CI	*P*
Pre-S-RT PSA	1.60	(1.01–2.52)	0.045	1.64	(1.03–2.61)	0.035
PSA nadir after RP	1.61	(0.01–259.95)	0.85	0.375	(0.002–88.09)	0.73
Period between RP and S-RT	1.00	(1.00–1.00)	0.68	1.00	(1.00–1.00)	0.28
Radiation dose (<65 Gy)	1.41	(0.92–2.15)	0.12	1.44	(0.94–2.22)	0.096
Age	1.02	(0.98–1.05)	0.31	1.02	(0.99–1.06)	0.24

PSA = prostate-specific antigen, S-RT = salvage radiation therapy, RP = radical prostatectomy, HR = hazard ratio, CI = confidence interval.

### Adverse events

The crude incidences of Grade 1, 2 and 3 acute adverse events were 51.1%, 7.0% and 0.5% for genitourinary (GU) events, and 36.6%, 22.0% and 0% for gastrointestinal (GU) events, respectively. No Grade 4 or higher acute adverse event was observed. The acute toxicities are listed in Table 4. Reported late adverse events are summarized in Table 5. The crude incidences of Grade 1, 2 and 3 late adverse events were 29.6%, 13.4% and 2.7% for GU events, and 15.6%, 4.3% and 0% for GI events, respectively. No Grade 4 or higher late toxicity was observed in either the acute or late phase.

**Table 4.** Incidences of acute adverse events

		Incidence (%)				
		Grade 0	Grade 1	Grade 2	Grade 3	Grade 4
GU	Miction pain	82.3	17.7	0	0	0
toxicity	Incontinence	87.6	11.3	1.1	0	0
	Pollakisuria/urgency	48.4	45.7	5.9	0	0
	Retention/obstruction	91.4	8.1	0	0.5	0
	Any GU event	41.4	51.1	7.0	0.5	0
GI	Proctitis	70.4	29.0	0.5	0	0
toxicity	Rectal bleeding	80.1	15.6	4.3	0	0
	Perianal mucositis	58.6	21.5	19.9	0	0
	Any GI event	41.4	36.6	22.0	0	0

GU = genitourinary, GI = gastrointestinal.

**Table 5.** Incidences of late adverse events

		Incidence (%)				
		Grade 0	Grade 1	Grade 2	Grade 3	Grade 4
GU	Miction pain	93.0	7.0	0	0	0
toxicity	Incontinence	68.8	23.1	7.5	0.5	0
	Pollakisuria/urgency	80.6	17.2	2.2	0	0
	Retention/obstruction	91.4	5.9	1.6	1.1	0
	Hematuria	82.3	12.9	3.8	1.1	0
	Any GU event	54.3	29.6	13.4	2.7	0
GI	Proctitis	90.3	9.1	0.5	0	0
toxicity	Rectal bleeding	83.3	15.1	1.6	0	0
	Perianal mucositis	91.4	5.9	2.7	0	0
	Any GI event	80.1	15.6	4.3	0	0

GU = genitourinary, GI = gastrointestinal.

Incidences of acute GU, acute GI and late GI adverse events were significantly lower in patients treated with ≥5-field technique compared with those who irradiated with the 4-field technique (*P* value: <0.0001, 0.0063 and 0.0024, respectively) (Tables 6 and 7). On the other hand, incidences of late GU events were significantly higher in patients who received higher radiation doses (*P* = 0.028) (Table 7).

**Table 6.** Incidences of acute adverse events by radiation technique and total dose

		Incidence (%)					*P* value
		Grade 0	Grade 1	Grade 2	Grade 3	Grade 4	
Incidences of any acute GU events (%)	4 fields	24.0	64.6	10.4	1.0	0	**<0**.**0001**
	≥5 fields	60.2	37.5	2.3	0	0	
	≤65 Gy	43.6	50.0	5.5	0.9	0	0.44
	>65 Gy	38.2	53.9	7.9	0	0	
Incidences of any acute GI events (%)	4 fields	30.2	42.7	27.1	0	0	**0.0063**
	≥5 fields	52.3	30.7	17.0	0	0	
	≤65 Gy	44.5	35.5	20.0	0	0	0.27
	>65 Gy	36.8	38.2	25.0	0	0	

GU = genitourinary, GI = gastrointestinal.

**Table 7.** Incidences of late adverse events by radiation technique and total dose

		Incidence (%)					*P* value
		Grade 0	Grade 1	Grade 2	Grade 3	Grade 4	
Incidences of any late GU events (%)	4 fields	46.9	40.6	10.4	2.1	0	0.32
	≥5 fields	61.4	18.2	17.0	3.4	0	
	≤65 Gy	60.9	26.4	10.0	2.7	0	**0.028**
	>65 Gy	44.8	34.2	18.4	2.6	0	
Incidences of any late GI events (%)	4 fields	71.9	21.9	6.2	0	0	**0.0024**
	≥5 fields	89.8	8.0	2.2	0	0	
	≤65 Gy	80.0	15.5	4.5	0	0	0.95
	>65 Gy	80.3	15.8	3.9	0	0	

GU = genitourinary, GI = gastrointestinal.

## DISCUSSION

S-RT is recognized as the sole approach affording an opportunity of a cure to patients with localized prostate cancer who develop PSA recurrence after RP [6]. The principal purpose of S-RT is to reduce the PSA level to the limit of detection, and maintain PSA recurrence-free status. According to a recent publication, the PSA recurrence-free rates were generally 30–60% 3–6 years after S-RT [6]. King performed a systematic review of S-RT based on the data of 41 reports, including 5597 cases, and the average 5-year PSA control rate was 46.2% [9]. In Japan, Kinoshita reported a 5-year PSA recurrence–free rate of 42.2% [10]. Therefore, long-term PSA control can generally be expected in about half of all patients who receive S-RT. The result of a current surveillance study (5-year PRFS rate = 50.4%) is very consistent with those of previous reports, and it will be possible to conclude that long-term PSA control may be expected in about half of all patients treated with S-RT, in Japan, following PSA failure after RP.

Various predictors of PRFS have been reported, including the pathological T-stage at RP, seminal vesicle (SV) invasion, surgical margin status, Gleason's score (GS), the period elapsing between RP and PSA failure, combined hormonal therapy, PSA doubling time, radiation dose, and PSA level at the time of S-RT initiation (pre-S-RT PSA) [6]. Generally, higher T-stage, positive SV invasion, a higher GS, a shorter period between RP and PSA failure, a shorter PSA doubling time, a lower radiation dose, and a higher pre-S-RT PSA level, predict poor PRFS. However, considerable among-report inconsistencies are evident.

Among possible predictors of PRFS, the pre-S-RT PSA level has consistently been found to be significant. King, in a comprehensive systematic review of S-RT, found that only the pre-S-RT PSA level (*P* < 0.001) and the radiation dose (*P* = 0.052) were independent significant predictors of PRFS [6, 9]. The PFRS fell by an average of 2.6% for each incremental 0.1 ng/ml of PSA level at the time of initiation of S-RT. This study affords Level 2a evidence for initiation of S-RT at the lowest possible PSA level. The results of our current study are in line with those of King. In the present study, the 5-year PRFS rates were 57.5% and 40.5% for patients who received S-RT at PSA values of 0.3 ng/ml or lower, and those commenced on S-RT at PSA levels over 0.3 ng/ml, respectively (*P* = 0.027).

We believe this finding is very important, because it finds immediate application in daily clinical practice. It is not in fact difficult to commence S-RT in routine practice as early as possible after PSA failure is observed, to (possibly) improve PRFS. In this sense, the pre-SRT PSA level is a very practicable predictive factor that can be used in daily clinical practice. On the other hand, most other possible predictive factors, including pathological T-stage, SV invasion, surgical margin status, and GS, are based on pre-operative or operative data. If a patient develops PSA failure after RP, it is usually difficult to decide not to offer S-RT to those with unfavorable predictions, because S-RT is the sole definitive treatment for cases who develop PSA recurrence after RP, and not all patients with poor predictors fail to benefit from S-RT.

Another possible means of improving PRFS after S-RT may be dose escalation. In the work of King, radiation dose was another independent predictor of PRFS [9]. S-RT doses in the range 60–70 Gy lie in the steep region of the sigmoid dose–response curve; a dose of 70 Gy was associated with 54% PRFS compared with only 34% for 60 Gy. However, no consensus has yet been reached on the optimal radiation dose for S-RT. In the present work, the radiation dose did not predict PRFS. This may be attributable to the fact that most patients (94%) in our series were treated with doses of 60–70 Gy; only six cases (3%) received 70 Gy or higher. The impact of dose escalation during S-RT delivered in Japan should be explored in future.

Toxicities associated with S-RT were very limited in our series of patients, in agreement with previously published data [6]. In our series, no Grade 4 or higher acute or late toxicity (either GU or GI) was observed. The incidences of Grade 3 toxicities were only 0.5% and 0% (acute GU and GI toxicities) and 2.7% and 0% (late GU and GI toxicities), respectively.

Adjuvant radiotherapy (A-RT) is also strongly recommended to patients with adverse pathological findings after RP (i.e. SV invasion, positive surgical margins, and/or extracapsular extensions), based on three randomized trials, all of which found that A-RT was useful [3, 11–14]. No completed trial has directly compared S-RT with A-RT. Although several retrospective comparisons between the two approaches have been conducted, it is impossible to retrospectively determine whether either modality is superior to the other. This is because patients who would not have developed recurrences without further intervention were included in the A-RT group, whereas all patients receiving S-RT had actually suffered recurrences. Currently, two prospective randomized trials comparing A-RT with a ‘wait-and- see’ policy following S-RT initiation at early trigger points (PSA > 0.1 or 0.2 ng/ml) are ongoing [15, 16].

Although S-RT affords an ∼50% chance of further long-term PSA control in patients who exhibit PSA recurrence after RP, no extra survival benefit thereof has been proven in comparison with hormonal therapy (HT) alone. The JCOG 0401 study was designed to answer this question [2]; however, the study is not yet complete. Even if the survival outcomes of the S-RT- and HT-alone groups are shown to be comparable, S-RT would still have the advantage of affording an HT-free chance in about half of all patients experiencing recurrence. Therefore, we believe that S-RT will play a useful role in patients who develop PSA failure after RP.

In conclusion, hormonal therapy is combined with S-RT in ∼40% of cases in Japan. S-RT is commenced when the PSA level attains ∼0.3 ng/ml (on average). The 5-year PRFS and CFFS rates of cases treated via S-RT alone were 50.1% and 90.1%, respectively. Incidences of acute GU, acute GI and late GI events were significantly lower in patients treated with 3D-CRT with five portals or more, when compared with those treated with the 4-field technique. A PSA value of 0.3 ng/ml was a significant cut-off predicting PRFS.

## FUNDING

This work was partly supported by the Japanese Radiation Oncology Study Group (JROSG). Funding to pay the Open Access publication charges for this article was provided by the grants and endowments of Kyoto University (#200040700171).
